# Risk factors for morbidity and mortality of bloodstream infection in patients undergoing hemodialysis: a nested case–control study

**DOI:** 10.1186/1756-0500-7-882

**Published:** 2014-12-07

**Authors:** Dayana Fram, Mônica Taminato, Vinicius Ponzio, Silvia Regina Manfredi, Cibele Grothe, Ruth Ester Assayag Batista, Angélica Belasco, Dulce Barbosa

**Affiliations:** School of Nursing, Escola Paulista de Enfermagem, Universidade Federal de São Paulo (School of Nursing, Federal University of São Paulo - EPE/UNIFESP), R. Napoleão de Barros 754, São Paulo, SP ZIP 04024-002 Brazil; Infection Control Unit, Instituto da Criança e Instituto de tratamento de câncer infantil, Faculdade de Medicina, Universidade de São Paulo (Institute of the Child and institute of Childhood Cancer Treatment, School of Medicine, University of São Paulo - ITACI/FMUSP), Av. Dr. Enéas Carvalho de Aguiar 647, São Paulo, SP ZIP 05403-000 Brazil; Division of Infectious Diseases, Escola Paulista de Medicina, Universidade Federal de São Paulo (Paulista School of Medicine, Federal University of São Paulo - EPM/UNIFESP), R. Napoleão de Barros, 715, 7° andar, São Paulo, ZIP 04024-002 Brazil; Division of Dialysis, Hospital do Rim e Hipertensão, Fundação Oswaldo Ramos (Kidney and Hypertension Hospital, Foundation Oswaldo Ramos - HRIM/FOR), R. Pedro de Toledo 282, São Paulo, SP ZIP 04039-030 Brazil

**Keywords:** Mortality, Hospitalization, Risk factor, Bacteremia, Bloodstream infection, *Staphylococcus aureus*, Hemodialysis

## Abstract

**Background:**

Infection is the leading cause of morbidity and the second most frequent cause of mortality among patients on renal replacement therapy. A major morbid event in this population is hospitalization because of infection. The aim of this study was to investigate the risk factors for morbidity and mortality related to bloodstream infection (BSI) among patients on hemodialysis.

**Results:**

Risk factors for morbidity and mortality related to BSI in patients on hemodialysis were investigated retrospectively by nested case–control, from January 2010 to June 2013. Patients were divided into two groups: those who progressed to hospitalization or death due to BSI (Group 1) and those who developed BSI, but did not progress to the same outcome (Group 2). Data were collected through consultation of patient records. For statistical analysis, logistic regression was used. There were 32 patients in Group 1 and 61 in Group 2. Logistic regression verified that, for each year of age, the chance of death or hospitalization for BSI increased 1.05 times [95% confidence interval (CI): 1.02–1.09]. Patients with BSI caused by *Staphylococcus aureus* had an 8.67 times higher chance of progressing to death or hospitalization (95% CI: 2.5–30.06). The isolation of multiresistant microorganisms in blood cultures of hemodialysis patients increased morbidity and mortality by 2.75 times (95% CI: 1.01–7.48).

**Conclusion:**

Independent risk factors for morbidity and mortality among patients after developing BSI during hemodialysis were: age, blood culture positive for *S. aureus*, and antibiotic resistance. Control measures to prevent microbial dissemination, primarily the multiresistant ones, should be intensified in this population. More studies are needed to standardize specific measures not yet classically standardized, such as collection of surveillance culture samples, contact precautions, and decolonization.

## Background

Infection is the leading cause of morbidity and the second most frequent cause of mortality among patients on renal replacement therapy [1] _._

North American data show that rates of bloodstream infection (BSI) in hemodialysis patients range from 0.5 to 27.1/100 patients/month, depending on the type of access used [2]. More recent studies demonstrated a reduction in the incidence of BSI in this population from 1.09 to 0.89/100 patients/month and from 2.04 to 0.75/100 patients/month after implementation of specific control measures [3, 4].

According to the US Renal Data System, one of the principal morbidity events in patients receiving renal replacement therapy is hospitalization because of infection, which increased by 43% between 1993 and 2011, contrasting with hospitalization for complications of venous access that were found to decrease by 57% [1]. The same study showed that the mortality rate at the end of the first year of hemodialysis decreased by 38% for cardiovascular diseases and 50% for infectious diseases from 2000 to 2010. All-cause mortality fell from 440 to 201 deaths/1000 patients/year, and for infectious diseases, the mortality rate decreased from 43 to 19.4 deaths/1000 patients/year [1].

Despite all measures to prevent BSI specifically in hemodialysis patients, this event continues to have a significant impact on the progression of these patients [5–8].

Most authors have shown that, among the microorganisms isolated in blood cultures of hemodialysis patients, 28–65% are Gram positive, and *Staphylococcus aureus* is the most frequent, and ∼ 45% are Gram negative [9–11]. Gram-positive microorganisms, mainly *S. aureus*, are associated with higher rates of mortality; primarily when resistant to methicillin (methicillin-resistant *S. aureus*; MRSA). The presence of septic shock and polymicrobial infections are related to higher mortality in this population [12, 13].

Several studies [14, 15] have examined the main risk factors for the development of BSI. The results of these studies have led to improved preventive measures, which may have caused a decrease in morbidity and mortality in this population. However, few studies [11] have analyzed the risk factors for morbidity and mortality in patients with end-stage renal disease (ESRD) who have already developed this complication.

The occurrence of adverse events, such as mortality and hospitalization, and frequent exposure to invasive procedures in the population susceptible to ESRD, motivated the present study, which aimed to identify risk factors for morbidity and mortality related to BSI.

## Methods

### Ethical aspects

The study received approval from the Committee on Ethics in Research of the Universidade Federal de São Paulo.

### Design, period and location of study

This was a retrospective nested case–control study, which investigated the prevalence of BSI during January 2010 to June 2013. This study was developed in an outpatient hemodialysis unit of the Hospital do Rim e Hipertensão da Fundação Oswaldo Ramos, São Paulo, Brazil. About 400 patients attend the dialysis program every month. There were 353 patients in the dialysis program during the study; 221 (63%) were on hemodialysis and 132 (37%) on peritoneal dialysis. This institution follows rigorous norms for the completion of patient records.

### Patients

We included only hemodialysis patients with ESRD, independent of treatment duration, aged ≥18 years, with positive blood culture, and conforming to specific criteria from the US Centers for Disease Control and Prevention (CDC) [16]. The blood cultures were processed according to the recommendations of the Clinical and Laboratory Standards Institute (CLSI) [17].

The patients were identified through a review of the reports of the microbiology laboratory, and only the first episode of BSI was considered. From a total of 221 patients on hemodialysis, 93 (42.1%) had BSI and met the inclusion criteria, and were divided into two groups. Patients who progressed to hospitalization or death were classified as Group 1. Patients who did not have the same progression were classified as Group 2. Hospitalization was considered to be related to BSI when patients were hospitalized for treatment of BSI, and death was considered related to BSI when it occurred within 15 days after microbiological diagnosis of infection [18, 19].

### Study protocol and data collection

After selecting the patients, data collection was conducted through consultation of the medical records. The following sociodemographic characteristics were recorded: age, sex, race, and educational level. The following clinical variables were recorded: comorbidity (defined by physician diagnosis reported in the medical records at patient admission to the hospital for hypertension, cardiovascular diseases, diabetes mellitus and other diseases); previous treatment for ESRD (patients may have undergone more than one type of treatment before the study, e.g., conservative treatment, peritoneal dialysis, hemodialysis and transplantation); body mass index (BMI) [20]; number of transfusions; use of previous antimicrobial agents; adequate empiric antibiotic therapy; previous hospitalization; number of previous hospitalizations. We also recorded variables related to hemodialysis (length of treatment, type, location and length of current venous access), and number of previous venous accesses), average values of laboratory tests, microorganisms isolated in blood culture, antibiotic resistance profile, and death. The variables were recorded for up to 6 months before BSI.

Empirical therapy was considered adequate when the microorganisms isolated in blood culture were sensitive to the antimicrobial agent used [11, 21]. The microorganisms were considered to be multiresistant when they showed resistance to at least one agent in three or more antimicrobial classes. *Staphylococcus* was considered multiresistant when it was not susceptible to methicillin (methicillin-resistant *S. aureus*; MRSA), and *Enterococcus* when it showed resistance to vancomycin [22, 23].

### Statistical analysis

A descriptive analysis was conducted of the patients who were hospitalized or progressed to death due to BSI (Group 1) versus patients who did not progress to these outcomes (Group 2). Sociodemographic, clinical and laboratory variables, and parameters related to treatment were considered. The data were presented using absolute frequencies and percentages, means, standard deviations and medians (25th–75th percentile), when appropriate. The primary outcome was death or hospitalization related to BSI (morbidity and mortality). The association between the primary outcome and the categorical variables was tested with the χ^2^ test, Fisher’s exact test, or the likelihood ratio. The association between continuous variables and the primary outcome was determined using Student’s *t* test, Mann–Whitney test and analysis of variance, as appropriate.

Univariate logistical regression was used to investigate the relationship of each independent variable with the dependent variable (death or hospitalization due to BSI). The variables with p ≤ 0.05 were selected for further analyses. The independent variables tested in univariate analyses were: sociodemographic characteristics, type and characteristics of hemodialysis access, laboratorial variables, and microbiological aspects of the microorganisms isolated. Multiple logistic regression was used to determine the independent variables that continued to be associated with morbidity and mortality. To include the variables in the regression model, the forward stepwise method was used. The odds ratios (ORs) were calculated with the respective 95% confidence intervals (CIs).

SPSS version 19.0 was used for statistical analysis (Chicago, IL USA).

## Results

There were 221 patients in the hemodialysis program during the study period and 93 (42.1%) met the inclusion criteria for the study. Of these, 32 (34.4%) progressed to death within 15 days of infection or were hospitalized for treatment of the infection (Group 1). Among the 32 patients, seven (22.0%) died, and 25 (78.0%) were hospitalized for treatment of BSI. Seventy-one patients (65.6%) did not have the same outcomes (Group 2).

Table 1 shows the sociodemographic and clinical characteristics of the patients evaluated. There was no significant difference between the groups, except for the variable of age (p = 0.028), which was significantly higher in Group 1.

**Table 1:** **Sociodemographic characteristics and clinical variables of the patients**

	Group 1	Group 2	
	n = 32	n = 61	p
Age	62 ± 17.8	53 ± 17.5	0.027
Sex			
Male	16 (50.0)	25 (41.0)	0.405
Female	16 (50.0)	36 (59.0)	
Race			
White	22 (69.0)	34 (55.7)	0.223
Non-white	10 (31.0)	27 (44.3)	
Educational level			
No education	10 (31.2)	15 (24.6)	0.757
Elementary	12 (37.5)	20 (32.8)	
High school	7 (21.9)	18 (29.5)	
Higher education	3 (9.4)	8 (13.1)	
Comorbidity			
Hypertension	30 (93.8)	49 (80.3)	0.127
Cardiovascular diseases	8 (25.0)	12 (19.7)	0.552
Diabetes mellitus	15 (47.0)	18 (29.5)	0.096
Other^a^	12 (37.5)	20 (32.8)	0.650
Previous treatment	20 (62.5)	43 (70.5)	0.434
Type of previous treatment			
Conservative	14 (43.8)	28 (46.0)	0.843
Peritoneal dialysis	6 (18.8)	15 (24.6)	0.522
Hemodialysis	5 (15.6)	13 (21.3)	0.510
Transplant	5 (15.6)	11 (18.0)	0.770
Body mass index (kg/m^2^)	24.8 ± 4.7	24.0 ± 4.4	0.374
Transfusions	1 (3.1)	10 (16.4)	0.090
No. of transfusions	1 (-)	2 ± 2.7	
Use of previous antimicrobials	15 (47.0)	19 (31.1)	0.135
Adequate empiric antibiotic therapy	24 (75.0)	51 (83.6)	0.318
Previous hospitalization	7 (21.9)	12 (19.7)	0.802
No. of previous hospitalizations	1.4 ± 0.5	1.5 ± 1.5	0.297

Values expressed as mean ± SD or n (%).

^a^Hypothyroidism, hyperthyroidism, neoplasia, or systemic lupus erythematosus.

There was no significant difference between the groups for variables related to hemodialysis (Table 2).

**Table 2:** **Variables related to hemodialysis treatment**

	Group 1	Group 2	
	n = 32	n = 61	p
Length of hemodialysis treatment (mo)	16 (4.5–0.5)	11 (3.0–0.8)	0.771
Type of current access			
AVF	12 (37.5)	15 (24.6)	0.097
CVC (long duration)	20 (62.5)	42 (68.9)	
CVC (short duration)	0 (-)	4 (6.6)	
Location of current venous access			
Jugular vein	17 (53.1)	42 (68.9)	0.308
Subclavian vein	4 (12.5)	4 (6.6)	
Upper arm	11 (34.4)	15 (24.6)	
Duration of current venous access			
1–30 d	6 (18.8)	15 (24.6)	0.533
30–180 d	9 (28.1)	21 (34.4)	
> 180 d	17 (53.1)	25 (41.0)	
Previous venous access (6 mo)	8 (25.0)	23 (37.7)	0.217
No. of previous accesses	1.1 ± 0.38	1.2 ± 0.39	0.853

Values expressed in mean ± SD, n (%) or median (25th–75th percentile).

AVF: arteriovenous fistula.

Table 3 presents the results of laboratory tests for each group. The average leukocyte count in Group 1 was significantly higher compared with Group 2.

**Table 3:** **Average values of laboratory tests for each group**

	Group 1	Group 2	
	n = 32	n = 61	p
Urea (mg/dL)	140 (45.1)	147 (31.6)	0.399
Creatinine (mg/dL)	8.4 (2.7)	9.3 (2.8)	0.152
Kt/V	1.4 (0.3)	1.4 (0.3)	0.439
Alkaline phosphatase (U/L)	74.5 (65.3-46.0)	82.3 (70.0-47.5)	0.241
Ferritin (mg/L)	705 (385.9)	573 (314.3)	0.094
Leukocyte (cells/mm^3^)	9038 (3083)	7582 (2362)	0.017
Hemoglobin (g/dL)	11.1 (1.4)	10.6 (1.5)	0.123
Hematocrit (%)	33.7 (4.6)	32 (4.9)	0.105
Albumin (g/dL)	3.7 (3.4-3.1)	3.7 (3.5-2.9)	0.425
Anti-HCV negative	31 (96.9)	61 (100)	0.165
Anti- HBs negative	30 (93.8)	57 (93.44)	0.954

Values expressed in mean ± SD, n (%) or median (25th–75th percentile).

HBs, hepatitis B surface antigen; HCV, hepatitis C virus; Kt/V, parameter used for measurement of the adequacy of hemodialysis treatment.

Table 4 shows the microorganisms isolated in blood culture. Among the microorganisms isolated from patients in Group 1, Gram-positive bacteria represented 84.3% (n = 27), and 55.6% were multiresistant, and Gram-negative bacteria accounted for 15.7% (n = 5) with 80% showing multiple resistance. The most frequent microorganism was *S. aureus* (50%; n = 16), and 43.8% of these were multiresistant. There were no fungi isolated in Group 1. Among the microorganisms isolated from patients in Group 2, 67.2% (n = 41) were Gram-positive bacteria and 41.5% were multiresistant, and 31.2% (n = 19) were Gram-negative bacteria, and 15.8% were multiresistant. Fungi accounted for 1.6% of the isolates.

**Table 4:** **Microorganisms isolated in blood cultures and profile of resistance**

	Group 1			Group 2		
	n = 32			n = 61		
	MR	S	Total	MR	S	Total
Gram positive						
*S. aureus*	7 (43.8)	9 (56.2)	16 (50.0)	5 (33.3)	10 (66.7)	15 (24.6)
Others^a^	8 (72.7)	3 (27.3)	11 (34.3)	12 (46.2)	14 (53.8)	26 (42.6)
Total	15 (55.6)	12 (44.4)	27 (84.3)	17 (41.5)	24 (58.5)	41 (67.2)
Gram negative						
Enterobacteriaceae	3 (75.0)	1 (25.0)	4 (12.5)	1 (11.1)	8 (88.9)	9 (14.8)
Non-fermenters	1 (100.0)	0 (-)	1 (3.2)	2 (20.0)	8 (80.0)	10 (16.4)
Total	4 (80.0)	1 (20.0)	5 (15.7)	3 (15.8)	16 (84.2)	19 (31.2)
Fungi						
*Candida albicans*	0 (-)	0 (-)	0 (-)	0 (-)	1 (100.0)	1(1.6)

Values expressed as mean ± SD or n (%).

^a^ *Streptococcus acidominimus*, *Streptococcus agalactiae*, *Streptococcus anginosus*, *Streptococcus bovis*, *Streptococcus pyogenes*,*Staphylococcus simulans*, *Staphylococcus warneri*, *Staphylococcus epidermidis*, *Staphylococcus capitis*, coagulase-negative *Staphylococcus*, *Staphylococcus haemolyticus*, *Staphylococcus lugdinensis and Enterococcus faecalis*.

MR: multiresistant, S: sensitive.

Table 5 shows the statistical analysis of the isolated microorganisms. The variables *S. aureus* and multiresistant microorganisms were statistically significant (p ≤ 0.05) in the initial analysis and were selected for logistic regression.

**Table 5:** **Initial statistical analysis of the microorganisms isolated in the groups**

	Group 1	Group 2	p
	n = 32	n = 61	
Gram staining			
Positive	27 (84.4)	41 (67.2)	0.095
Negative	5 (15.6)	19 (31.1)	
Comparison between microorganisms			
*S. aureus*	16 (50.0)	15 (24.6)	0.014
Other microorganisms	16 (50.0)	46 (75.4)	
*S. aureus*	16 (50.0)	15 (24.6)	0.066
Other Gram-positive organisms	11 (34.4)	26 (42.6)	
Enterobacteriaceae	4 (12.5)	9 (14.8)	0.327
Non-fermenters	1 (3.1)	10 (16.4)	
Resistance profile			
Multiresistant	19 (59.4)	20 (32.8)	0.014
Sensitive	13 (40.6)	41 (67.2)	
Gram-positive multiresistant	15 (46.9)	17 (27.9)	0.255
Gram-positive sensitive	12 (37.5)	24 (39.3)	
*S. aureus* multiresistant	7 (21.9)	5 (8.2)	0.552
*S. aureus* sensitive	9 (28.1)	10 (16.4)	

The results of logistic regression analysis are shown in Table 6. In the univariate analysis, the variables significantly associated with morbidity and mortality were: age, *S. aureus* and multiresistant microorganisms. All of the significant variables in the univariate analysis were independent risk factors for morbidity and mortality among patients in the multivariate analysis. For each year of age increase, the chance of death or hospitalization due to BSI increased 1.05 times (95% CI: 1.02–1.09). Patients with BSI caused by *S. aureus* had 8.67 times more chance of progression to death or hospitalization (95% CI: 2.5–30.06) in relation to BSI caused by other microorganisms. The isolation of multiresistant microorganisms in the blood cultures of hemodialysis patients increased morbidity and mortality by 2.75 times (95% CI: 1.01–7.48) when compared with the isolation of sensitive organisms.

**Table 6:** **Univariate and multivariate logistic regression analysis**

	n	Simple logistic regression		Multiple logistic regression	
		OR (95% CI)	p	OR (95% CI)	p
Age	93	1.03 (1.00–1.06)	0.031	1.05 (1.02–1.09)	0.004
Microorganisms	93				
*S. aureus* (31) X other microorganisms (62)		3.07 (1.24–7.58)	0.015	8.67 (2.5–30.06)	<0.001
Resistance profile of microorganisms	93				
Multiresistant (39) X sensitive (54)		3.00 (1.24–7.26)	0.015	2.75 (1.01–7.48)	0.047

X: versus.

## Discussion

The present study evaluated the risk factors for morbidity and mortality in patients with BSI undergoing hemodialysis.

Rojas et al. [24] conducted a retrospective cohort study in the nephrology department of a Spanish teaching hospital to evaluate the risk factors for mortality and other unfavorable outcomes in patients with BSI. Age was one of the risk factors associated with mortality (p = 0.02). Hospitalization was not evaluated, and the study also included all patients undergoing renal replacement therapy and not just specifically hemodialysis as in the current study. Another retrospective study, conducted in a Swiss university hospital, evaluated outcomes of two groups of older patients on hemodialysis, divided by age range (60–69 and ≥70 years) and did not find any significant differences in relation to all-cause mortality (p = 0.07) and hospitalization (p = 0.06) [25].

The current study showed that age was related to morbidity and mortality, and for every annual increase in age, the chance of death or hospitalization due to BSI increased 1.05 times (95% CI: 1.02–1.09).

*S. aureus* is associated with high mortality rates, especially MRSA [12, 13]. Engemann et al. [26] conducted a prospective study in a North American university hospital over a 7-year period. They followed 210 patients undergoing hemodialysis who were hospitalized for BSI with *S. aureus*. All-cause mortality rate was 19% (n = 40) compared with 10.5% (n = 22) for BSI with *S. aureus*.

Danese et al. [27] conducted a retrospective cohort study in the US to evaluate first hospitalization for BSI in patients in the first year of hemodialysis. They showed that BSI caused by *S. aureus*, when compared with BSI caused by other microorganisms, was associated with 17% higher mortality (OR: 1.17, 95% CI: 1.08–1.26).

In the present study, BSI with *S. aureus* was related to an increase of 8.67 times in the chance of patients progressing to death or hospitalization (95% CI: 2.5–30.06) when compared with BSI caused by other microorganisms.

The majority of the studies evaluated morbidity and mortality among patients with BSI caused by MRSA or methicillin-sensitive *S. aureus* (MSSA). In a retrospective study, FitzGerald et al. [13] demonstrated a significant difference between the proportion of deaths that occurred in patients with BSI caused by MRSA compared with MSSA (p < 0.01).

Conterno et al. [18] performed a nested case–control study to identify risk factors for mortality for BSI caused by *S. aureus*, and showed a mortality risk 4.2 times higher among patients who developed MRSA bacteremia when compared with the patients that developed bacteremia due to MSSA. Surveillance data on MRSA from the CDC showed that 90% of patients on hemodialysis or peritoneal dialysis, who developed BSI caused by MRSA, were hospitalized, and the rate of MRSA-related mortality was 17% [28]. Another study that evaluated the progression of hemodialysis patients who developed BSI caused by MRSA or MSSA demonstrated similar mortality between the two groups (30.9 and 32.1%, respectively) [29].

Data from the present study demonstrated that multiresistant microorganisms isolated in blood culture from hemodialysis patients increased morbidity and mortality by 2.75 times (95% CI: 1.01–7.48) when compared with the isolation of sensitive microorganisms. There are no data in the literature comparing morbidity and mortality among patients with BSI caused by any multiresistant microorganism and patients who developed BSI caused by susceptible microorganisms.

According to several authors [11, 14, 15], the use of short-duration central venous catheter (CVC) and a new vascular access are important risk factors for BSI. However, the aim of our study was to evaluate risk factors for morbidity and mortality in hemodialysis patients with BSI. The presence, time of use, and type of catheter were not risk factors for the outcomes studied. The use of short-duration CVC is not common in patients with ESRD, given that only four patients included in our study used this device and, all belonged to Group 2. Long-duration CVC was used in both groups (20 in Group 1 and 42 in Group 2), also without statistical significance between the groups.

We demonstrated that risk factors for morbidity and mortality among patients with BSI on hemodialysis were: age, isolation of *S. aureus*, and isolation of a resistant microorganism. The result reinforces the need to implement specific control measures to decrease the spread of multiresistant microorganisms in this population.

The control of the dissemination of *S. aureus* in hemodialysis patients, along with general measures of infection control, requires other specific measures. For example, Sesso et al. [30] showed in a prospective randomized study that administration of mupirocin at the CVC insertion site in hemodialysis patients significantly reduced the risk of colonization and bacteremia by *S. aureus*. In a recent systematic literature review and meta-analysis, Taminato et al. [31] demonstrated that mupirocin is effective for reduction of BSI caused by *S. aureus*.

Preventive measures that are already standardized should be intensified and controlled through vigilance, including: surveillance of infection, education of health professionals, hand washing, use of personal protective equipment, environmental cleaning, maximization of barriers to insertion of CVC, use of skin antisepsis before catheter insertion and dressing changes, inspection of the CVC insertion site, protection of CVC, and replacing the catheter-site dressing when it becomes damp, loosened or soiled, [4, 5, 8, 32].

The positive deviance (PD) to engage the staff has been used to reduce BSI in outpatient hemodialysis centers [33]. PD consists of a social and behavioral change process to deal with matters. This process is based on the fact that in institutions there are groups or individuals with different (deviant) practices that achieve positive results when compared with co-workers with the same resources [34]. PD has been successfully applied in different countries to reduce infection by multiresistant organisms, surgical site infection, and improve hand hygiene adherence [35–37]. Strategies that are not yet classically recommended, such as collection of surveillance cultures, decolonization and contact barriers, require more studies to demonstrate their effectiveness [38, 39].

With regard to the limitations of the current study, these were inherent in the retrospective character of the data collection. However, the medical records of the patients in our institution were completed with great care, due to the characteristics of being a service dedicated to the treatment of chronic renal patients, which follows the directives of the organization. Additionally, there were standardized protocols for the performance of blood cultures in these patients and it was unlikely that these events were underdiagnosed during the study.

## Conclusions

Risk factors for morbidity and mortality among patients with BSI undergoing hemodialysis were: age, isolation of *S. aureus*, and isolation of resistant microorganisms. The results of this study emphasize the importance of investment in prevention and control of the dissemination of microorganisms; mainly multiresistant microorganisms that were associated with greater morbidity and mortality in this population. Process measures can be a useful tool for the control of already standardized protocols. More studies are needed to standardize additional measures of prevention.

## Authors’ information

DF: nurse, MSc and PhD; MT: nurse, MSc, PhD and post-doctorate (EPE/UNIFESP); VP: physician, MSc and doctoral student (EPM/UNIFESP); SRM: nurse, MSc and doctoral student (EPE/UNIFESP); CG: nurse, PhD and post-doctorate (EPE/UNIFESP); REAB: nurse, MSc, PhD and adjunct professor (EPE/ UNIFESP); AB: nurse, MSc, PhD and adjunct professor (EPE/UNIFESP); and DB: nurse, PhD, MSc and associate professor, livre docente (EPE/UNIFESP).
